# Modulation of Cellular Levels of Adenosine Phosphates and Creatine Phosphate in Cultured Primary Astrocytes

**DOI:** 10.1007/s11064-023-04039-y

**Published:** 2023-10-19

**Authors:** Gabriele Karger, Julius Berger, Ralf Dringen

**Affiliations:** 1 Faculty 2 (Biology/Chemistry), Centre for Biomolecular Interactions Bremen, University of Bremen, P.O. Box 330440, 28334 Bremen, Germany; 2 Centre for Environmental Research and Sustainable Technologies, University of Bremen, Bremen, Germany

**Keywords:** Astrocytes, ATP, Creatine, Metabolism, Mitochondria

## Abstract

Adenosine triphosphate (ATP) is the main energy currency of all cells, while creatine phosphate (CrP) is considered as a buffer of high energy-bond phosphate that facilitates rapid regeneration of ATP from adenosine diphosphate (ADP). Astrocyte-rich primary cultures contain ATP, ADP and adenosine monophosphate (AMP) in average specific contents of 36.0 ± 6.4 nmol/mg, 2.9 ± 2.1 nmol/mg and 1.7 ± 2.1 nmol/mg, respectively, which establish an adenylate energy charge of 0.92 ± 0.04. The average specific cellular CrP level was found to be 25.9 ± 10.8 nmol/mg and the CrP/ATP ratio was 0.74 ± 0.28. The specific cellular CrP content, but not the ATP content, declined with the age of the culture. Absence of fetal calf serum for 24 h caused a partial loss in the cellular contents of both CrP and ATP, while application of creatine for 24 h doubled the cellular CrP content and the CrP/ATP ratio, but did not affect ATP levels. In glucose-deprived astrocytes, the high cellular ATP and CrP contents were rapidly depleted within minutes after application of the glycolysis inhibitor 2-deoxyglucose and the respiratory chain inhibitor antimycin A. For those conditions, the decline in CrP levels always preceded that of ATP contents. In contrast, incubation of glucose-fed astrocytes for up to 30 min with antimycin A had little effect on the high cellular ATP content, while the CrP level was significantly lowered. These data demonstrate the importance of cellular CrP for maintaining a high cellular ATP content in astrocytes during episodes of impaired ATP regeneration.

## Introduction

The brain is metabolically highly active and uses around 20% of the glucose and oxygen consumed by the body [1, 2]. The oxygen consumed in brain is needed mainly for ATP regeneration by mitochondrial oxidative phosphorylation as this process provides the large amounts of metabolic energy required to fuel the information transfer between neurons [3]. Nevertheless, astrocytes which are essential partners of neurons in brain [4–6], also need substantial amounts of ATP for their many essential functions. Important ATP-consuming astrocytic processes are the maintenance of the cellular membrane potential [7, 8], neurotransmitter uptake [9], glutamine synthetase-catalyzed amidation of glutamate to glutamine [10], ATP-driven export processes [11] as well as the synthesis of energy stores such as glycogen [12] and fatty acids [2, 13]. The synthesis and continuous availability of high ATP levels are highly important to fuel all astrocytic contributions to the normal brain functions.

ATP is regenerated in astrocytes by phosphorylation of ADP mainly by cytosolic glycolysis and by mitochondrial oxidative phosphorylation [14, 15]. Both processes are used for ATP regeneration in astrocytes, and each appears to have sufficient capacity to, at least in part, compensate for an impairment of the other one. This is demonstrated by the surprising ability of cultured astrocytes to maintain a high cellular ATP concentration after glucose-deprivation or after treatment with a mitochondrial inhibitor in the presence of glucose [15] and to survive an impairment of the respiratory chain as glycolytic cells [16, 17]. However, astrocytes are rapidly depleted of their cellular ATP, if both the glycolytic substrate chain phosphorylation plus the mitochondrial oxidative phosphorylation are impaired [15, 18–22].

Cultured astrocytes contain a high cellular ATP concentration of around 7 mM [15]. In contrast, such cultures contain only low amounts of ADP and AMP [23–27]. This leads to high values for the calculated adenylate energy charge (AEC: ([ATP] + 0.5 [ADP])/([ATP] + [ADP] + [AMP])) [28, 29] of around 0.9 in untreated cultured astrocytes [24, 26, 27].

Creatine phosphate (CrP) serves in many cell types as rapidly available and quickly mobilizable buffer of bound high energy-rich phosphate groups that has the exclusive function of supporting rapid phosphorylation of ADP to ATP via creatine kinase (CrK) [30–33]. Cellular CrP is generated by phosphorylation of creatine by CrK which is present in high specific activity in cultured astrocytes [34]. The cellular creatine that serves as substrate of CrK can be synthesized in astrocytes from the precursor amino acids glycine, arginine and methionine [35, 36] or is derived from uptake of exogenous creatine [37]. Due to the high AEC [24, 26, 27] and the high activity of CrK [34] most of the cellular creatine should be present in astrocytes as CrP. Indeed, cultured astrocytes contain specific CrP levels which have been reported to be similar or even higher than those reported for ATP [38–41]. To our knowledge only few studies have reported conditions that modulate CrP levels in astrocytes. Under ischemic conditions, both ATP and CrP levels decline in cultured astrocytes with similar velocity, reaching 50% of the initial values after around 6 h of incubation [40]. Interestingly, exposure of astrocytes to ammonia or octanoate [39] or to glutamate [42] lowers the cellular CrP levels, while the high cellular ATP contents remains unaltered. These results support the view that CrP serves also in astrocytes as temporary energy buffer to prevent cellular ATP depletion.

In order to investigate the interplay between different adenosine phosphates and CrP in astrocytes, we have investigated the basal contents of adenosine phosphates and CrP in cultured astrocytes and have explored treatments which may affect those levels. Here we report that the specific CrP contents, but not the ATP contents, as well as the ratio of CrP/ATP decline with the age of astrocyte cultures, while supplementation of the medium with creatine almost doubled CrP contents and CrP/ATP ratio. In addition, impairment of glycolysis by 2-deoxyglucose and of oxidative phosphorylation by antimycin A caused a rapid decline in cellular CrP levels that preceded the decline in ATP contents. In contrast, for glucose-fed astrocytes antimycin A hardly affected the high cellular ATP content but lowered the CrP level severely during a 30 min incubation. These data demonstrate the importance of CrP in astrocytes as rapidly mobilizable energy buffer that helps to maintain a high cellular ATP concentration, especially during episodes of impaired mitochondrial ATP production.

## Materials and Methods

### Materials

Sterile cell culture materials, unsterile 96-well plates and black microtiter plates were purchased from Sarstedt (Nümbrecht, Germany). Fetal calf serum (FCS), adenylate kinase, AMP, antimycin A, creatine, creatine kinase and 2-deoxyglucose (2DG) were obtained from Sigma-Aldrich (Steinheim, Germany; RRID:SCR_008988). ADP, Dulbecco’s modified Eagles medium (DMEM with 25 mM glucose, catalog number 52100-021) and penicillin G/streptomycin sulfate solution were from Thermo Fisher Scientific (Schwerte, Germany). The Cell Titer Glo^®^ 2.0 ATP Assay Kit was purchased from Promega (Walldorf, Germany; RRID:SCR_006724). Bovine serum albumin, dimethyl sulfoxide (DMSO) and perchloric acid were from AppliChem (Darmstadt, Germany; RRID:SRC_005814). ATP and pyruvate kinase were purchased from Roche Diagnostics (Mannheim, Germany; RRID:SCR_001326). All other basal chemicals were obtained from Sigma-Aldrich (Steinheim, Germany), Roth (Karlsruhe, Germany), Riedel-de Haën (Seelze, Germany) or Fluka (Buchs, Switzerland).

### Astrocyte Primary Cultures

Wistar rats were obtained from Charles River Laboratories (Sulzfeld, Germany; RRID:SCR_003792). Animals were treated in accordance to the State of Bremen, German and European animal welfare acts. Primary astrocyte cultures were prepared from the brains of newborn rats as previously described in detail [43]. Of the harvested cell suspension, 300,000 viable cells were seeded per well of 24-well dishes in 1 mL culture medium (90% DMEM containing 25 mM glucose, 44.6 mM sodium bicarbonate, 1 mM pyruvate, 20 U/mL penicillin G, 20 µg/mL streptomycin sulfate, supplemented with 10% FCS). The cultures established from the seeded cells remained in the wells without sub-culturing in the humidified atmosphere a Sanyo CO_2_ incubator (Osaka, Japan) containing 10% CO_2_. The culture medium was renewed every seventh day and one day prior to experiments. For the current study, confluent primary astrocyte cultures of an age between 14 and 28 days after seeding were used. Astrocyte-rich primary cultures were frequently characterized by immunocytochemical staining for cells positive for the astrocyte marker protein glial fibrillary acidic protein. These cultures are strongly enriched in astrocytes and contain only low numbers of contaminating other types of glial cells [43–45].

### Experimental Incubation of Astrocytes

To test for the consequences of a preincubation of astrocytes with serum or creatine, the culture medium of astrocyte primary cultures in wells of 24-well dishes was replaced by 1 mL DMEM (containing 25 mM glucose, 44.6 mM sodium bicarbonate, 1 mM pyruvate, 20 U/mL penicillin G, 20 µg/mL streptomycin sulfate) that had been supplemented with or without 10% FCS and/or 1 mM creatine. After 24 h incubation at 37 °C in the humidified atmosphere of an incubator with 10% CO_2_ supply, the cells were washed twice with 1 mL ice-cold (4 °C) phosphate-buffered saline (PBS; 10 mM potassium phosphate buffer pH 7.4 containing 150 mM NaCl) and lysed for quantification of adenosine phosphates and CrP.

To test for the short-time consequences of glucose deprivation and/or the application of metabolic inhibitors, astrocyte primary cultures in wells of 24-well dishes were washed twice with 1 mL pre-warmed (37 °C) glucose-free incubation buffer (IB; 145 mM NaCl, 20 mM HEPES, 5.4 mM KCl, 1.8 mM CaCl_2_, 1 mM MgCl_2_, 0.8 mM Na_2_HPO_4_, pH adjusted with NaOH to 7.4 at 37 °C) and subsequently incubated for up to 30 min at 37 °C in the humidified atmosphere of a CO_2_-free incubator in 250 µL glucose-free IB that had been supplemented with the given substrates and/or inhibitors. After the incubation periods given, the incubation media were harvested to test for potential cell damage by measuring the activity of extracellular lactate dehydrogenase (LDH), while the cells were washed twice with 1 mL ice-cold (4 °C) PBS and lysed for quantification of adenosine phosphates and CrP.

### Determination of Cellular Contents of Adenosine Phosphates and Creatine Phosphate

Perchlorate lysates of cultured astrocytes were used to quantify the cellular contents of adenosine phosphates and CrP. Briefly, the cultures were washed twice with 1 mL ice-cold (4 °C) PBS and lysed in 400 µL of ice-cold 0.5 M HClO_4_ on ice for 5 min. The lysates were collected and 2 M KOH was added to neutralize the lysates. After centrifugation at 12,100×g for 5 min to precipitate KClO_4_, the supernatant was harvested for quantification of adenosine phosphates and CrP. ATP in the lysate was determined as recently described [15, 46] using a luciferine-luciferase-based luminometric assay.

ADP, AMP and CrP in the lysates were converted by enzymatic reactions to ATP that was subsequently quantified by the luminometric ATP assay. To convert CrP to ATP in astrocyte lysates, a reported method [47] was adapted. Ten µL neutralized lysate was diluted with 190 µL 70 mM Tris/acetate buffer pH 7.75 and 50 µL reaction mixture (3 µM ADP, 1 mM MgCl_2_ in 70 mM Tris/acetate buffer pH 7.75 without (only for ATP quantification) or with 5 U creatine kinase/50 µL (quantification of CrP plus ATP)) were added. After 60 min incubation at 37 °C, the phosphate group of CrP had been completely transferred to ADP to generate ATP.

To convert AMP and/or ADP into ATP in astrocyte lysates, a reported method [48] was adapted. Ten µL sample of the neutralized 400 µL lysate was diluted with 40 µL 70 mM Tris/acetate buffer pH 7.75 and subsequently with additional 50 µL of reaction mixtures that contained 3 mM phosphoenolpyruvate, 20 mM MgCl_2_, and 70 mM Tris/acetate buffer pH 7.75 without (for ATP quantification) or with 2 U/50 µL pyruvate kinase (for quantification of ADP plus ATP) or 2 U/50 µL pyruvate kinase plus 9 U/50 µL adenylate kinase (for quantification of AMP plus ADP plus ATP). After 45 min incubation at room temperature ADP and AMP had been completely phosphorylated to ATP. Application of additional ATP for AMP phosphorylation by adenylate kinase plus pyruvate kinase is not required as already minute amounts in the lysates of initial ATP or of ATP generated from the initial ADP by pyruvate kinase are sufficient to convert AMP quantitatively to ATP under the assay conditions used (data not shown).

Finally, 50 µL of the neutralised lysate samples or ATP standards (ATP quantification) or 50 µL of the reactions used to convert the molecules of interest into ATP (see above) were diluted in wells of a black 96-well plate with 50 µL of the ATP detection reagent (Cell Titer Glo^®^ 2.0 ATP Assay Kit) to start the luciferase reaction. After 30 min of incubation, the luminescence signal was recorded by a Fluoroskan Ascent FL chemiluminescence plate reader (Thermo Fisher Scientific, Bremen, Germany). ATP values for the samples were calculated by using the linear calibration curve generated from the values obtained for the ATP standards (0–1000 nM to measure CrP and ATP and 0–2000 nM to measure ATP, ADP and AMP). The amounts of CrP, ADP and AMP were calculated by subtracting from the total ATP content determined per well (cellular ATP plus ATP generated by enzymatic conversion of AMP, ADP or CrP to ATP) the initial ATP contents (for CrP and ADP quantification) or the ATP plus ADP contents (for AMP quantification). Specific contents were calculated by normalizing the respective values determined to the initial cellular protein content of the cultures.

### Determination of Cell Viability and Protein Content

The extracellular activity of LDH in 10 µL media samples harvested after a given incubation was compared with the initial cellular LDH activity to test for potential toxicity as previously described [43]. The initial cellular protein content per well was determined by the Lowry method [49] using bovine serum albumin as standard protein.

### Data Presentation and Statistical Analysis

The data shown in figures and tables represent means ± standard deviations (SDs) of values obtained from three or more experiments that were each performed in duplicates on independently prepared astrocyte cultures. For data sets derived from at least 5 independent experiments normality distribution was tested for by the Kolmogorov–Smirnov test. For data from less than 5 independent experiments statistical analysis was done under the assumption of normal distribution. Analysis for statistical significance between groups of data was performed by ANOVA followed by the Bonferroni post-hoc test and the calculated level of significance compared to the indicated control condition is given by *p < 0.05, **p < 0.01 and ***p < 0.001. Differences between two groups of data were tested for statistical significance by t test and the calculated level of significance compared to the control condition is given by ^#^p < 0.05, ^##^p < 0.01 and ^###^p < 0.001. p > 0.05 was considered as not significant.

## Results

### Culture Age Dependency of the Cellular Contents of Adenosine Phosphates and CrP in Cultured Astrocytes

To determine the basal contents of adenosine phosphates and CrP in cultured astrocytes and to investigate a potential dependency of these contents on the age of the culture, specific values that had been determined in a total of 22 experiments (performed on 16 independently prepared cultures) were analysed (Table 1). The average specific contents of ATP, ADP and AMP were found to be 36.0 ± 6.4 nmol/mg, 2.9 ± 2.1 nmol/mg, and 1.7 ± 2.1 nmol/mg protein, respectively. The average sum of all three adenylates was 40.7 ± 6.6 nmol/mg and the average AEC accounted to 0.92 ± 0.04 (Table 1). The average specific CrP content of the cultures was found to be 25.9 ± 10.8 nmol/mg and the ratio of CrP to ATP in cultured astrocytes was calculated to be 0.74 ± 0.28 (Table 1).

**Table 1:** Basal levels of adenosine phosphates and CrP in cultured astrocytes after incubation for 24 h in culture medium without (control) or with 1 mM creatine

	Control	1 mM creatine
Protein content (µg/well)	133 ± 26	135 ± 31
ATP content (nmol/mg)	36.0 ± 6.4	33.0 ± 5.8
ADP content (nmol/mg)	2.9 ± 2.1	2.3 ± 1.8
AMP content (nmol/mg)	1.7 ± 2.1	1.6 ± 1.3
Sum of adenosine phosphates (nmol/mg)	40.7 ± 6.6	39.4 ± 4.0
AEC	0.92 ± 0.04	0.93 ± 0.02
CrP content (nmol/mg)	25.9 ± 10.8	51.3 ± 17.3^###^
CrP/ATP ratio	0.74 ± 0.28	1.56 ± 0.44^###^

The data presented are means ± SD of values that were obtained in 22 (control) or 13 (creatine) individual measurements on cell lysates that had been obtained from 16 (control) or 10 (creatine) independently prepared cultures. The significance of differences (unpaired t test) between data for incubations without and with creatine is indicated by ^###^p < 0.001

**Fig. 1:**
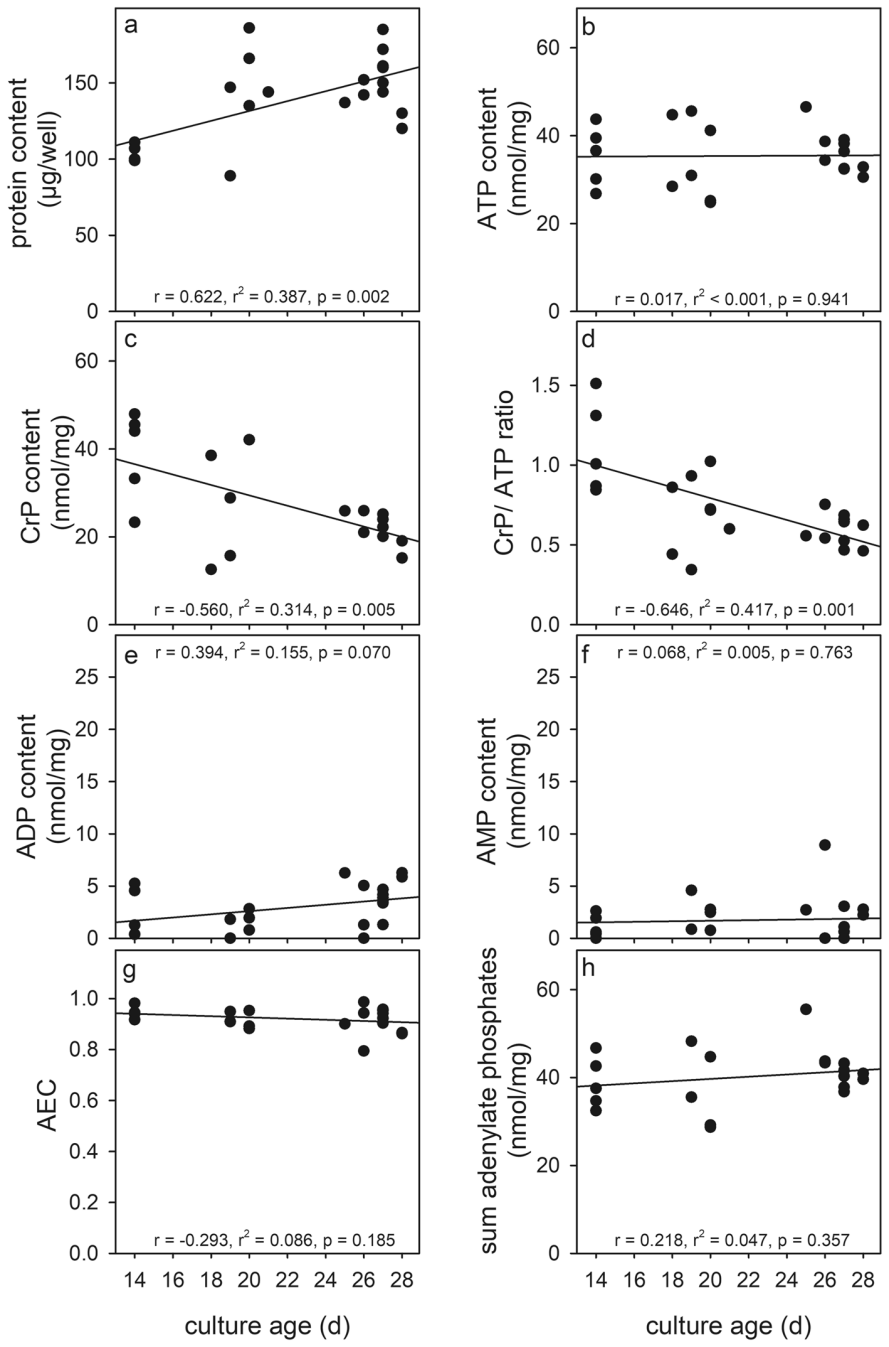
Basal levels of adenosine phosphates and CrP in cultured astrocytes. The cellular protein content (**a**) as well as the specific contents of ATP (**b**), CrP (**c**), ADP (**e**) and AMP (**f**) were determined for untreated astrocyte primary cultures of the given culture ages. In addition, the ratio of CrP to ATP contents (**d**), the AEC (**g**) as well as the sum of contents of the three adenosine phosphates (**h**) were calculated. The data shown were obtained in 22 experiments performed on 16 independently prepared cultures. Lines in the panels are derived from first order regression analyses of the data obtained and the respective correlation parameters are given in the individual panels

With increasing culture age, the protein content per well (Fig. 1a) increased slightly, while the specific contents of ATP (Fig. 1b), ADP (Fig. 1e) and AMP (Fig. 1f) as well as the calculated AEC (Fig. 1g) and the sum of adenosine phosphates (Fig. 1h) remained almost constant. For individual experiments substantial differences to the average values (Table 1) were observed, but no obvious age-dependent alterations of the specific contents of the three adenosine phosphates were found (Fig. 1). In contrast, the specific CrP content (Fig. 1c) as well as the ratio of CrP to ATP (Fig. 1d) declined with increasing culture age, explaining the substantial SDs obtained for the average values determined for CrP content and for the CrP/ATP ratio (Table 1).

### Consequences of Creatine Supplementation on the Contents of CrP and Adenosine Phosphates in Cultured Primary Astrocytes

**Fig. 2:**
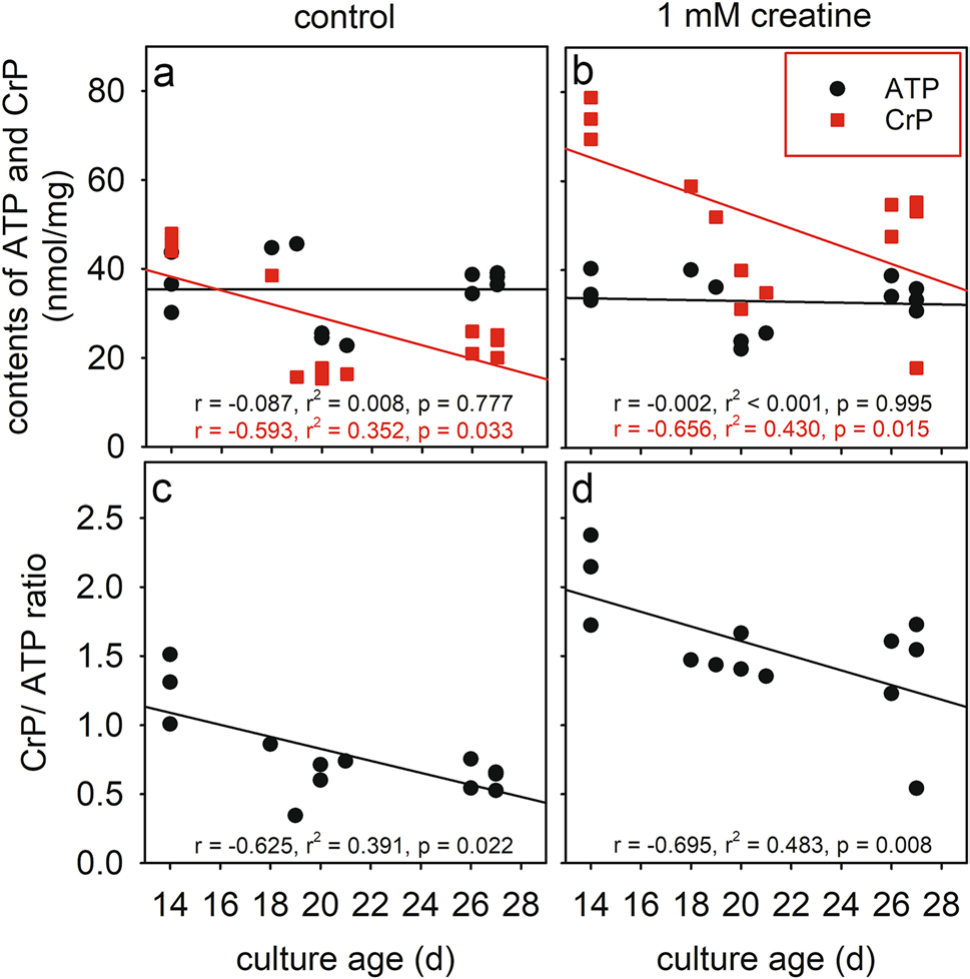
Modulation of CrP levels by an application of creatine to cultured astrocytes. The cultures were incubated for 24 h in fresh culture medium without (**a**, **c**) or with (**b**, **d**) 1 mM creatine before the specific cellular contents of ATP and CrP (**a**, **b**) were determined and correlated to the age of the culture. In addition, the ratio of CrP to ATP contents were calculated (**c**, **d**). The data shown were obtained in a total of 13 experiments performed on 10 independently prepared astrocyte cultures. Lines in the panels are derived from first order regression analyses of the data obtained and the respective correlation parameters are given in the individual panels

To test whether creatine supplementation may affect the cellular levels of CrP and ATP, the specific contents of ATP and CrP in cultured astrocytes that had been incubated for 24 h in culture medium without or with 1 mM creatine were analyzed. These incubations did not lead to any obvious toxicity nor to an alteration in the morphology of the cells in the confluent astrocyte cultures (data not shown). While the average cellular ATP content was not affected by a preincubation with creatine (Table 1, Fig. 2a, b), the average specific CrP level in creatine-exposed astrocytes as well as the CrP/ATP ratio were doubled by the creatine treatment (Table 1). An increase in the specific CrP content of creatine-fed astrocytes by around 25 nmol/mg compared to respective control cells (incubation without creatine) was observed (Table 1) which was irrespective of the culture age (Fig. 2a, b) and caused a substantially increased CrP/ATP ratio in both young and older astrocyte cultures (Fig. 2c, d).

**Fig. 3:**
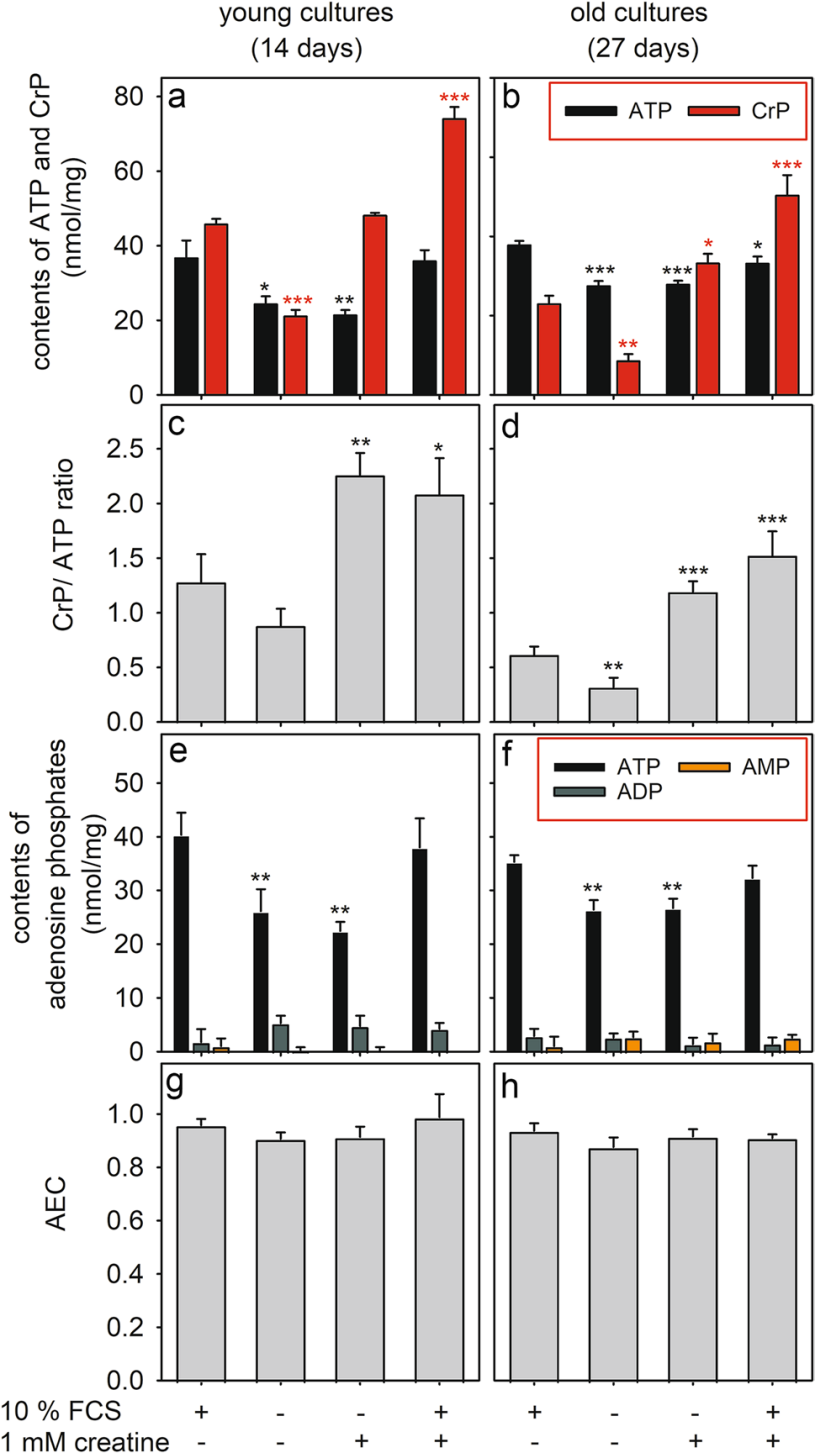
Comparison of young and old astrocyte cultures concerning their contens of CrP and adenosine phosphates. Astrocyte cultures of an age of 14 d (**a**, **c**, **e**, **g**) or 27 d (**b**, **d**, **f**, **h**) were incubated for 24 h in DMEM with or without 10% FCS and/or 1 mM creatine before the specific CrP and ATP contents (**a**, **b**, **e**, **f**) as well as the specific ADP and AMP contents (**e**, **f**) were determined. In addition, the ratio of CrP to ATP contents (**c**, **d**) as well as the AEC (**g**, **h**) were calculated. The data shown are means ± SD of values obtained in three experiments performed on independently prepared cultures. The average protein contents were 100 ± 1 μg/well (14 d-old) and 157 ± 6 μg/well (27 d-old). The significance of differences (ANOVA) of data compared to those of cells that had been incubated in DMEM with 10% FCS is indicated by *p < 0.05, **p < 0.01 and ***p < 0.001

To investigate the importance of FCS and creatine for the maintenance of astrocytic CrP and ATP levels, 14 d-old and 27 d-old cultures were incubated for 24 h in DMEM with or without 10% FCS and/or 1 mM creatine. Absence of FCS significantly lowered cellular ATP and CrP contents in young (Fig. 3a, e) and older cultures (Fig. 3b, f) and also lowered to some extend the CrP/ATP ratios (Fig. 3c, d), demonstrating that presence of serum is required to maintain the normal high contents of ATP and CrP in cultured astrocytes. Supplementation of DMEM with creatine prevented the decline in cellular CrP levels that was caused by serum-deprivation, but not the decline in cellular ATP contents (Fig. 3a, b). Finally, the presence of creatine in serum-containing medium strongly increased the specific CrP contents (Fig. 3a, b) as well as the CrP/ATP ratios (Fig. 3c, d) in both young and older astrocytes cultures, compared to an incubation in serum-containing DMEM, but did not affect ATP contents (Fig. 3a, b). None of the conditions applied caused any significant alteration in the cellular levels of ADP or AMP (Fig. 3e, f) nor in the AEC (Fig. 3g, h), compared to the control condition (presence of 10% FCS).

### Modulation of CrP and ATP Levels in Astrocytes by Glucose Deprivation, 2DG Application and/or Inhibition of the Respiratory Chain

ATP is regenerated in astrocytes mainly by glycolysis and mitochondrial respiration [15]. To test how the absence or the presence of glucose and/or the inactivation of glycolysis and/or mitochondrial respiration may affect cellular ATP and CrP contents, astrocyte cultures were incubated for up to 30 min with or without glucose or with the glycolysis inhibitor 2DG [50, 51] in the absence or the presence of 10 µM of the respiratory chain inhibitor antimycin A [52]. None of the conditions used compromised the cell viability as demonstrated by the absence of any significant increase in extracellular LDH activity (Table 2).

**Table 2:** Viability of primary astrocytes after various treatments

Treatment	Extracellular LDH activity (%)	n
Control	5 ± 3	9
10 µM antimycin A	2 ± 1	9
5 mM glucose	4 ± 2	6
5 mM glucose + 10 µM antimycin A	6 ± 2	6
10 mM 2-deoxyglucose	4 ± 3	3
10 mM 2-deoxyglucose + 10 µM antimycin A	4 ± 3	3

As indicator for potential loss in membrane integrity the extracellular LDH activity was determined after 30 min incubations of astrocytes in incubation buffer containing the compounds indicated. The LDH activity is given as percent of the initial cellular LDH activity (100% = 153 ± 36 nmol/(min × well)). The data shown are means ± SD of values obtained in experiments that had been performed on n independently prepared cultures

**Fig. 4:**
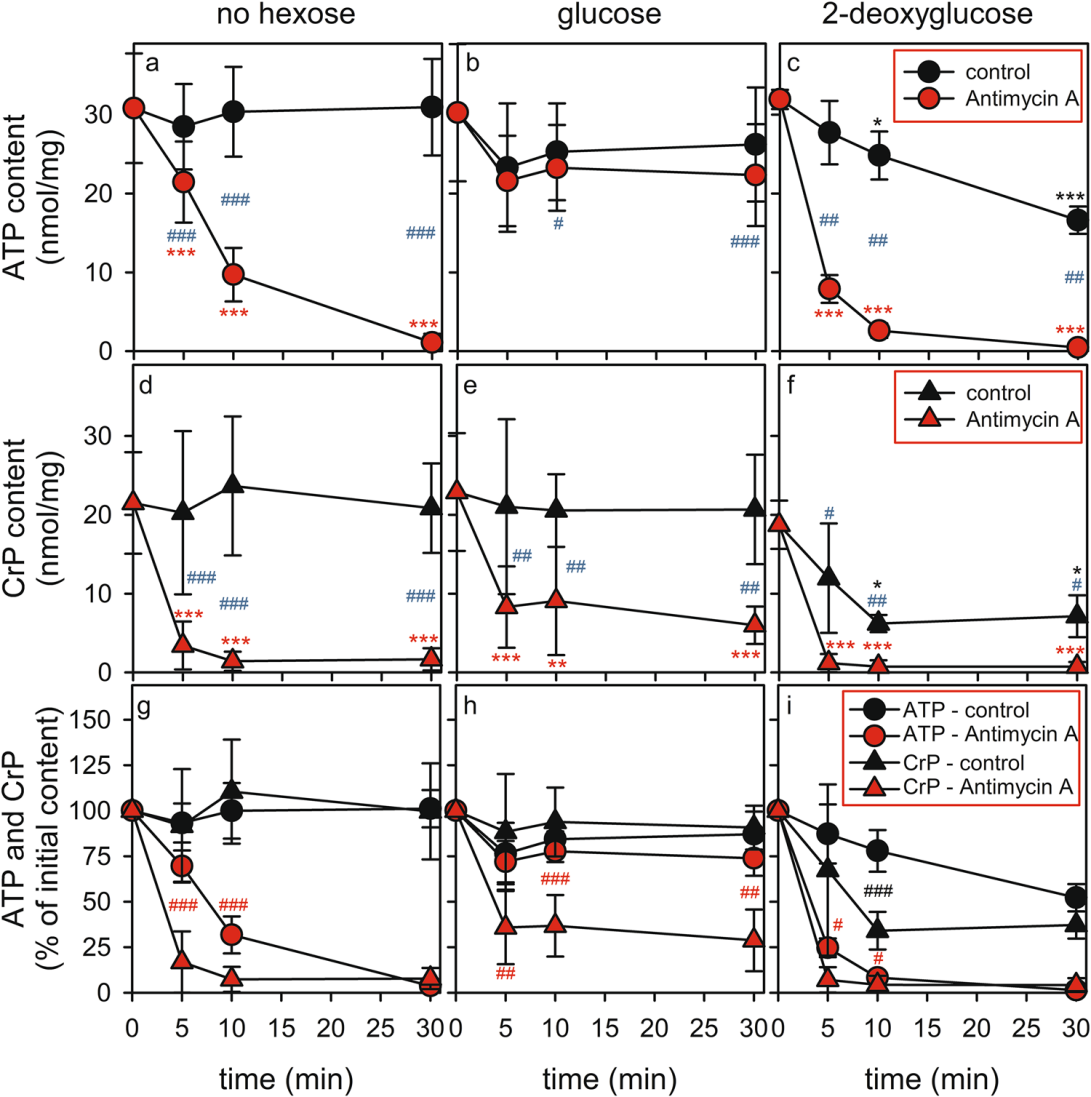
Modulation of CrP and ATP contents by glucose deprivation, 2DG application and/or antimycin A exposure in primary astrocyte cultures. The cells were incubated in incubation buffer containing no hexose (**a**, **d**, **g**), 5 mM glucose (**b**, **e**, **h**) or 10 mM 2DG (**c**, **f**, **i**) in the absence or the presence of 10 µM antimycin A for up to 30 min before the cellular contents of ATP (**a**–**c**) and CrP (**d**–**f**) were determined. Panels **g**–**i** give the cellular contents of ATP and CrP as percent of the respective initial contents at the onset of the experiment. The data shown are means ± SD of values obtained in nine (no hexose), six (glucose) and three (2DG) experiments performed on independently prepared cultures. The average initial protein contents of the cultures were 139 ± 25 μg/well (no hexose), 147 ± 26 μg/well (glucose) and 124 ± 19 μg/well (2DG). The significance of differences (ANOVA) of data compared to the initial contents is indicated by *p < 0.05, **p < 0.01 and ***p < 0.001 in the colours of the respective conditions (black for control, red for antimycin A-treatment). The significance of differences (paired t test) between data for incubations without and with antimycin A (in panels **a**–**f**) are indicated by blue hashes and those between the percental data for ATP and CrP for the respective conditions (in panels g-i) by black (control) or red (antimycin A-treatment) hashes with ^#^p < 0.05, ^##^p < 0.01 and ^###^p < 0.001

During incubations of astrocytes with or without glucose in the absence of antimycin A neither the specific ATP content (Fig. 4a, b) nor the specific CrP content (Fig. 4d, e) were significantly altered compared to the cellular contents at the onset of the incubation. In contrast, incubations in the presence of antimycin A strongly affected cellular ATP and CrP levels (Fig. 4). In glucose-deprived astrocytes, an incubation with antimycin A lowered cellular ATP levels within 10 min and 30 min to 33% and 4% of the initial content (Fig. 4a). The decline in cellular CrP was even more rapid for this condition (Fig. 4d) and preceded that of the decline in cellular ATP content (Fig. 4g). Already after 5 min of incubation CrP levels were found to be lowered by around 90% (Fig. 4d, g).

For glucose-fed astrocytes the presence of antimycin A lowered cellular ATP content only to a small extent (Fig. 4b), while a substantial loss of around 80% of the initial CrP content was observed during a 30 min incubation of glucose-fed astrocytes with antimycin A (Fig. 4e, h).

The presence of 2DG reduced the cellular content of ATP slowly by around 50% within 30 min (Fig. 4c), while the additional application of antimycin A severely accelerated the decline in cellular ATP in 2DG-treated astrocytes (Fig. 4c) compared to cells that had been incubated with antimycin A in the absence of 2DG (Fig. 4a). After exposure to 2DG the cellular CrP content was lowered within 10 min to around 34% of the initial value and did not further decline during longer incubations (Fig. 4f). For astrocytes that had been treated with 2DG plus antimycin A, already after 5 min of incubation CrP was hardly detectable (Fig. 4f). The decline in cellular CrP content in 2DG-treated astrocytes preceded that of the cellular ATP content for incubations without and with antimycin A (Fig. 4i).

### Modulation of the Contents of Adenosine Phosphates in Astrocytes by Glucose Deprivation, 2DG Application and/or Inhibition of the Respiratory Chain

**Fig. 5:**
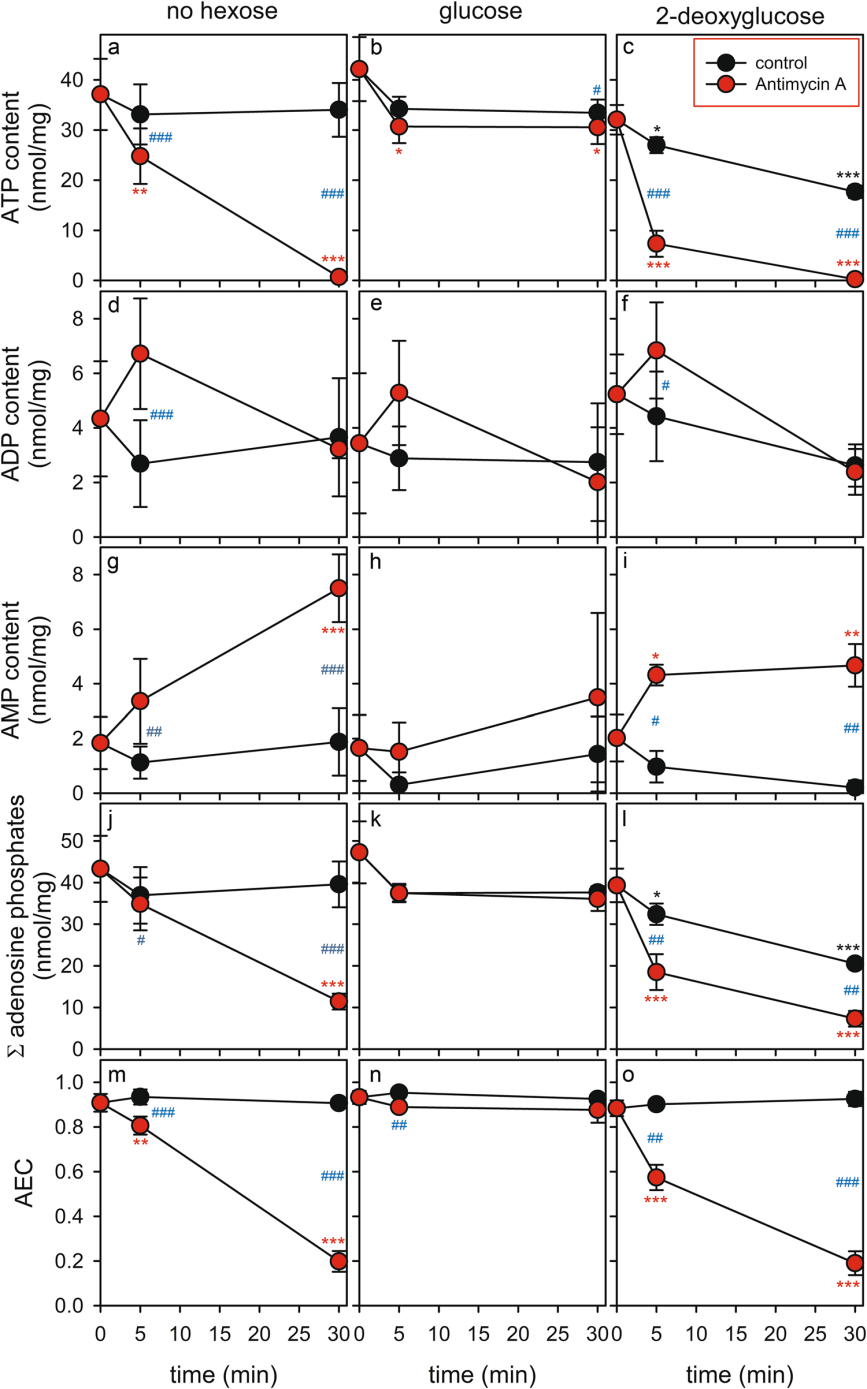
Modulation of the contents of adenosine phosphates by glucose deprivation, 2DG application and/or by antimycin A exposure in primary astrocyte cultures. The cells were incubated in incubation buffer containing no hexose (**a**, **d**, **g**, **j**, **m**), 5 mM glucose (**b**, **e**, **h**, **k**, **n**) or 10 mM 2DG (**c**, **f**, **i**, **l**, **o**) in the absence or the presence of 10 µM antimycin A for up to 30 min before the cellular contents of ATP (**a**–**c**), ADP (**d**–**f**) and AMP (**g**–**i**) were determined. In addition, the sum of all three adenosine phosphates (**j**–**l**) and the AEC (**m**–**o**) were calculated. The data shown are means ± SD of values obtained in six (no hexose) or three (glucose, 2DG) experiments performed on independently prepared cultures. The initial protein contents of the cultures were 128 ± 14 μg/well (no hexose), 126 ± 15 μg/well (glucose) and 124 ± 19 μg/well (2DG). The significance of differences (ANOVA) of data compared to the initial contents is indicated by *p < 0.05, **p < 0.01 and ***p < 0.001 in the colours of the respective conditions (black for control, red for antimycin A-treatment). The significance of differences (paired t test) between the data obtained for incubations with or without antimycin A is indicated by blue hashes with ^#^p < 0.05, ^##^p < 0.01 and ^###^p < 0.001

To test how the absence or the presence of glucose, the inactivation of glycolysis and/or the inhibition of mitochondrial respiration may affect cellular levels of ATP, ADP and AMP, cultured astrocytes were incubated for up to 30 min without or with glucose or 2DG in the absence or the presence of 10 µM antimycin A (Fig. 5). Incubations in the absence of antimycin A, irrespective of the absence or the presence of glucose, did not alter significantly the specific contents of ATP (Fig. 5a, b), ADP (Fig. 5d, e), AMP (Fig. 5g, h) nor the sum of adenosine phosphates (Fig. 5j, k) or the AEC (Fig. 5m, n). In contrast, for glucose-deprived astrocytes that had been exposed to antimycin A a rapid loss in cellular ATP content (Figs. 4a, 5a), a small transient increase in the cellular ADP content (Fig. 5d), a significant increase in the cellular AMP level (Fig. 5g) and a strong decline in the sum of the three adenosine phosphates (Fig. 5j) and in AEC (Fig. 5m) was observed.

For cells that had been exposed to 2DG (in the absence of antimycin A), a decline in the cellular level of ATP (Fig. 5c) and in the sum of the three adenylates (Fig. 5l) was observed, while the specific contents of ADP (Fig. 5f) and AMP (Fig. 5i) remained low and the AEC was not altered and maintained high (Fig. 5o). In contrast, a coincubation of astrocytes with 2DG plus antimycin A caused a rapid loss in cellular ATP content (Fig. 5c), a rapid transient increase in the cellular ADP content (Fig. 5f), a robust significant increase in the cellular AMP level (Fig. 5i) as well as a strong decline in the sum of adenosine phosphates (Fig. 5l) and in the AEC (Fig. 5o).

## Discussion

To investigate the interplay of ATP and CrP metabolism of astrocytes, we have used confluent astrocyte cultures of an age between 14 and 28 days. The average specific content of ATP in untreated astrocyte cultures was with 36.0 ± 6.4 nmol/mg in the range between 20 and 40 nmol/mg that has been reported previously for astrocyte cultures by several groups [15, 19, 21, 27, 46, 50]. The specific cellular ADP and AMP contents of untreated cultured astrocytes were very low compared to the ATP content, consistent with literature data for these adenosine phosphates [23–27] and with the reported high AEC of around 0.9 in astrocytes [24, 26, 27]. The average specific CrP content of cultured astrocytes was with 25.9 ± 10.8 nmol/mg similar to that of ATP as reported previously by other groups [38–41].

Comparison of cellular CrP and adenylate contents of astrocyte cultures of different ages revealed that the specific cellular CrP level declined with increasing culture age, while the specific contents of ATP, ADP and AMP as well as the AEC did not show any obvious age-dependent alterations. Some age-dependent declines in creatine [53] or CrP [53, 54] contents have previously been reported for muscle and brain tissue [55]. For cultured astrocytes, impaired cellular creatine synthesis [35, 36], increased creatinine formation [56] and/or impaired creatine uptake [37] may contribute to the observed age-dependent decline in the specific CrP content. However, as the application of creatine strongly increased the cellular CrP level by almost the same extent in young and older cultures, it can be assumed that an age-dependent impairment of cellular creatine uptake is unlikely to contribute to the observed decline in cellular CrP content with culture age.

Serum-deprivation lowered both ATP and CrP levels in cultured astrocytes, suggesting that serum contains compounds which support the maintenance of high cellular levels of both compounds. A loss in cellular ATP and CrP of astrocytes under such conditions is not unexpected as some cellular CrP is likely to disappear by spontaneous formation of creatinine [57] and as ATP can be exported from astrocytes [58, 59]. Serum components may be used by astrocytes to compensate for these losses. Indeed, serum contains creatine in concentrations of up to 500 µM [60, 61] which could serve as substrate for the active creatine uptake into astrocytes [37]. Similarly, ATP is present in serum [62] and products of the extracellular hydrolysis by astrocytes of this ATP [63] are likely to be taken up into the cells and serve as substrates for ATP synthesis via the purine salvage pathway [24, 64, 65].

Astrocytes are known to efficiently metabolize glucose and to produce ATP via glycolysis [66], but glucose-depletion did not affect the initial high cellular ATP content. This was expected for the short incubation periods used in our study (up to 30 min) as intracellular energy stores such as glycogen and fatty acids enable astrocytes to maintain a high ATP level in the absence of exogenous glucose for many hours by mitochondrial oxidative phosphorylation [15]. Also, the inhibition of mitochondrial respiration in the presence of glucose hardly affected cellular ATP levels, suggesting that upregulated glycolysis can at least for a short incubation period of up to 30 min compensate for an impairment in mitochondrial ATP regeneration. However, severe effects on the cellular ATP levels were observed, if both glycolysis and mitochondrial ATP regeneration had been compromised in cultured astrocytes, consistent with literature data [15, 18–22]. For such conditions a transient increase in the cellular ADP content was determined after 5 min incubation and a slower but stronger increase in the cellular AMP level during 30 min of incubation. The observed time-dependent changes in the cellular levels of adenosine phosphates are likely to be caused by the action of adenylate kinase that has been reported to be present in cultured astrocytes [34]. This enzyme transfers the terminal phosphate group of one ADP molecule to a second ADP molecule [67], thereby regenerating ATP and producing AMP. However, the sum of all three adenosine phosphates was found dramatically lowered in glucose-deprived and antimycin A-treated astrocytes, suggesting that a strong cellular accumulation of AMP which may strongly activate AMP-mediated signaling is partially prevented by subsequent AMP metabolism, for example by deamination of AMP to inosine monophosphate or by dephosphorylation of AMP to adenosine [68]. Further studies are now required to elucidate whether starved astrocytes indeed release AMP-derived metabolites such as adenosine and/or inosine monophosphate.

Astrocytic ATP content was slowly lowered after application of 2DG within 30 min to around 50% of the initial content, with the specific contents of ADP and AMP declining accordingly. This allowed the cells to maintain a high cellular AEC, despite a substantial loss in the sum of cellular adenosine phosphates. In contrast, inactivation of mitochondrial respiration in glucose-deprived and/or 2DG-treated astrocytes caused a rapid depletion of cellular ATP within minutes and the cells were unable to maintain a high AEC.

For all the conditions investigated in our study which caused a decline in cellular ATP contents we also observed a rapid loss in cellular CrP content that always preceded that of ATP. This observation supports the view that cultured astrocytes use their high CrP content as temporary buffer of high energy phosphate groups that is immediately used to regenerate ATP from accumulating ADP during periods of insufficient ATP regeneration by glycolysis or mitochondrial metabolism. The high specific activity of CrK (around 3 U/mg) that has been reported for cultured astrocytes [34] will enable these cells to rapidly regenerate ATP by phosphorylating ADP on the expense of their cellular CrP content, consistent with the view that CrK-mediated ATP regeneration is at least one order of magnitude faster than ATP regeneration by glycolysis and oxidative phosphorylation [69].

In the presence of glucose, an application of antimycin A for up to 30 min hardly affected the cellular levels of adenosine phosphates while the cellular content of CrP was strongly lowered by such a treatment. This observation suggests that under conditions of limited ATP regeneration by impaired mitochondrial respiration and accelerated glycolysis [15, 17] a high cytosolic ATP content and a high AEC are only maintained on the expense of the cellular CrP content. Similar results have been reported for an exposure of astrocytes to ammonia or octanoate [39] or to glutamate [42] which also resulted in lower cellular CrP levels while cellular ATP contents were maintained high.

In conclusion, a rapid decline of cellular CrP always preceded the decline in ATP content in cultured astrocytes under conditions that compromised cellular ATP regeneration by compromising glycolysis and/or mitochondrial oxidative phosphorylation. These data demonstrate the importance of cellular CrP as temporary and rapidly mobilizable energy buffer for maintaining a high cellular ATP content in astrocytes, especially during episodes of impaired mitochondrial ATP production. A supplementation with creatine doubled the cellular contents of CrP and the CrP/ATP ratio in astrocytes which may improve the ability of these cells to temporarily deal with situations of compromised metabolic ATP regeneration. Further studies are now required to elucidate beneficial consequences of a treatment of astrocytes with creatine and whether such processes may contribute to the wide range of reported health and therapeutical beneficial effects of a creatine supplementation [70–74].

## Data Availability

Enquiries about data availability should be directed to the authors.
